# Surveillance diagnostic algorithm using real-time PCR assay and strain typing method development to assist with the control of *C. auris* amid COVID-19 pandemic

**DOI:** 10.3389/fcimb.2022.887754

**Published:** 2022-08-31

**Authors:** Deisy A. Contreras, Margie A. Morgan

**Affiliations:** Clinical Microbiology Laboratory, Department Pathology and Laboratory Medicine, Cedars-Sinai Medical Center, Los Angeles, CA, United States

**Keywords:** *candida auris*, surveillance pcr, strain typing, epidemiology, algorithm

## Abstract

*Candida auris* continues to be a global threat for infection and transmission in hospitals and long-term care facilities. The emergence of SARS-CoV-2 has rerouted attention and resources away from this silent pandemic to the frontlines of the ongoing COVID-19 disease. Cases of *C. auris* continue to rise, and clinical laboratories need a contingency plan to prevent a possible outbreak amid the COVID-19 pandemic. Here, we introduce a two-tier *Candida auris* surveillance program that includes, first, a rapid qualitative rt-PCR for the identification of high-risk patients and, second, a method to analyze the isolated *C. auris* for strain typing using the Fourier-Transform Infrared spectroscopy. We have performed this two-tier surveillance for over 700 at-risk patients being admitted into our hospital and have identified 28 positive specimens (4%) over a 1-year period. Strain typing analysis by the IR spectrum acquisition typing method, supplemented by whole genome sequencing, has shown grouping of two significant clusters. The majority of our isolates belong to circulating African lineage associated with *C. auris* Clade III and an isolated strain grouping differently belonging to South Asian lineage *C. auris* Clade I. Low numbers of genomic variation point to local and ongoing transmission within the Los Angeles area not specifically within the hospital setting. Collectively, clinical laboratories having the ability to rapidly screen high-risk patients for *C. auris* and to participate in outbreak investigations by offering strain typing will greatly assist in the control of *C. auris* transmission within the hospital setting.

## Introduction

*Candida auris* continues to be a major public health threat, due to the unrelenting nosocomial spread in long-term and acute care medical facilities. This fungal pathogen causes a wide array of clinical presentations ranging from invasive nosocomial bloodstream to deep wound infections usually affecting the most vulnerable immunocompromised patient population resulting in high mortality rates (Du et al., 2020). The first incident of *C. auris* was in an unrecognized case of candidemia in South Korea dating back to as early as 1996 (Forsberg et al., 2019) followed by its first identified official report in 2009 in Japan (Satoh et al., 2009). To date, there are five different clades of *C. auris* that have been identified globally, in which four out of the five have been reported in the United States, attributing their introduction through international travel and medical tourism (Chow et al., 2018). In addition to its elusive nature and spread, this yeast exhibits resistance patterns in all the three widely used classes of antifungals including azoles, echinocandins, and polyenes, making it a critical antibiotic resistance threat in the US. The Centers for Disease Control and Prevention (CDC, 2019) stresses the need for the implementation of accurate diagnostic methods and infection prevention mitigation measures in US medical institutions.

While hospitals were preparing to combat this emerging yeast pathogen, the world was confronted by another novel agent causing a severe pneumonia-like illness, which was first identified in Wuhan, Hubei province, China in January 2020 as SARS-CoV-2 (Wu et al., 2020). This viral agent and succeeding variants were defined by increased transmissibility and high mortality rates, leaving patients in intensive care units supported on ventilators and immunosuppressive drugs vulnerable. Hospitals were soon met with an overwhelmingly need to focus all resources on preventing the spread of SARS-CoV-2, leaving them and their immune paralyzed patient population susceptible to secondary nosocomial infections such as those caused by *C. auris*. As the medical community learned to better treat and contain COVID-19 infections, hospitals were observing an increase in *C. auris* cases. By January 2021, cases of *C. auris* infections and transmission were at the frontline of most hospitals, specifically in those patients who have spent time in long-term acute care hospitals (LTACH). Containment of *C. auris* among patients, specifically residents of high-acuity long-term care facilities and ventilator-equipped skilled nursing facilities (vSNF), remains a critical public health priority, predominantly in the context of the current COVID-19 pandemic.

The Department of Public Health put out a health advisory for healthcare facilities alerting them of this need to contain nosocomial transmission rates of *C. auris*. The current guidelines called for active surveillance for the rapid identification of high-risk patients colonized with *C. auris* and implementation of infection control measures. Rapid identification could be achieved by the employment of highly sensitive real-time polymerase chain reaction (rt-PCR) platform. In addition to rapid identification of colonized patients, an alternative strain typing method was needed to aid in outbreak investigations within the clinical setting. In this context, the laboratory turned to spectroscopy-based technique, particularly the IR Biotyper (Bruker Daltonics, Germany), which utilizes mid-infrared (IR) radiation associated with Fourier transform IR (FTIR) spectroscopy. This direct IR radiation generates vibration patterns, primarily in the C-O stretching of biomolecules such as carbohydrates (800–1,300 cm^−1^) within the biochemical structure of these prokaryotic cell. These vibration patterns generate strain-specific absorbance fingerprints within the IR spectrum (Vatanshenassan et al., 2020), which can be used to differentiate among isolates. Literature has shown extensive reports illustrating the discriminatory power among both Gram-positive and Gram-negative bacteria, particularly *Listeria* spp., *Bacillus* spp., *Pseudomonas* spp., *Klebsiella* spp., and *Salmonella* spp., both at the genus and species levels (Quintelas et al., 2018; Hu et al., 2020; Rakovitsky et al., 2020; Cordovana et al., 2021; Mullie et al., 2022; Pascale et al., 2022; Wang-Wang et al., 2022). The IR Biotyper has proven to be an effective tool for microbial strain typing, specifically for those pathogens that are associated with nosocomial outbreaks within the hospital setting (Deidda et al., 2021; Lombardo et al., 2021; Guerro-Lozano et al., 2022; Hadas et al., 2022; Li et al., 2022; Passaris et. al., 2022).

In this study, we aim to discuss implementation for enhanced detection and further prevention of transmission of *Candida auris* through 1) active surveillance by rt-PCR and 2) strain typing by IR Biotyper. This two-tier diagnostic algorithm assesses the status of patients being admitted into the hospital for those individuals identified to be at highest risk for *C. auris* before being admitted to the hospital.

## Materials and methods

### Study design and participants

This study was conducted at a 900-bed tertiary care hospital and affiliate 200-bed community hospital in the coastal and westside communities of Los Angeles. Specimens were prospectively collected and *Candida auris* isolates were recovered from combined axilla/groin surveillance specimens testing positive by rt-PCR (n = 19) and from microbiological cultures from infections including wound (n = 2), respiratory (n = 4), and blood (n = 3). The surveillance rt-PCR assay was implemented as a tool to aid in the detection of *C. auris* from high-risk groups starting in August of 2021 for both our main campus and affiliate hospital. The high-risk criteria for patient inclusion in surveillance testing were established following discussion with our local public health department. Since initial studies, the microbiology laboratory has recovered an additional 28 C*. auris* isolates that were included in this study. *C. auris* isolates were recovered from critically ill patients with complicated clinical conditions that had a history of medical care in intensive care units and long-term care facilities. Patients ages range from 41 to 60 (n = 10), 61 to 70 (n = 6), and >70 (n = 12) with an equal distribution of both male and female patients.

### *C. auris* real-time surveillance PCR assay

The surveillance PCR assay utilizes the BioGX *Candida auris* rt-PCR reagents (BioGX, Inc., USA) for use with the BD MAX™ open testing system. The assay uses single vial, lyophilized ITS2 primer/probe set (Leach et al., 2018), which are used in conjunction with the BD MAX™ ExK DNA-3 extraction kit (BD Inc., USA). The assay extraction and PCR settings were used according to the manufacturer. In brief, the sample extraction parameters were set as follows: lysis heat time, temperature, sample tip height, and volume were all set to manufacturer default settings, with the exception, of sample volume that was set to 700 µl. The PCR parameters were set to the manufacturer recommendations with the 585/630 detector channel set for *C. auris* and the 680/715 detector channel for the sample processing control sequence from *Drosophila melanogaster* (GenBank, AC246436) with a default cross threshold setting of 200 for both fluorophore channels. The cycling conditions for the assay included an initial hold of 99°C for 300 s, followed by 40 cycles of 99°C for 10 s, 58°C for 30 s, and 70°C for 15 s. To avoid any type of amplicon or high-titer cross contamination, unidirectional workflow was established by keeping specimen preparation and reagent preparation separate.

### IR Biotyper strain typing

Any positive *C. auris* surveillance PCR specimen was inoculated on inhibitory mold agar (Becton Dickinson, USA) and CHROM^™^ Candida + auris (Hardy Diagnostics, USA) to isolate *C. auris.* Once isolate identification was confirmed to be *C. auris* using MALDI-TOF MS (Bruker Daltonics, USA), it was then plated on Sabouraud Dextrose (SabDex) agar (Becton Dickinson, USA) and incubated at 35°C for 24 h to prepare for strain typing. *Candida auris* isolates recovered from culture were directly inoculated on SabDex agar and incubated at designated standardized temperature and incubation period as stated above. As recommended by the manufacturer, two loopfuls using a 10 µl inoculation loop is harvested from the confluent portion of the yeast colony and added to a 1.5-ml suspension vial filled with 70 µl of freshly prepared 70% ethanol. According to the manufacturer, the amount of yeast colony does not need to be exact, but the pellet should be visible to ensure that there is enough biomass for processing. The suspension vials contain metal cylinders to aid in sample homogenization. The inoculated suspension vial is then vortexed for 10 min. Once initial resuspension of the organism is complete, and then, equal volume of deionized water is added to the suspension vial and vortexed for an additional 5 min. In parallel, IRTS1 and IRTS2 control vials are prepared by adding 90 µl of deionized water to each vial and mixing by vortex for 10 min and then adding undiluted ethanol in equal volume. The suspension was homogenized for an additional 5 min. To perform spectrum acquisition, 15 µl of the processed specimen was plated at a minimum plated in quintuplicates on the silicone sample plate and allowed to airdry on the silicone microtiter plate and allowed to air-dry. Spectra was recorded using an IR Biotyper spectrometer (Bruker Daltonik, USA). The recorded spectra were then visualized and processed by OPUS v8.2 software. To expand on differences between spectra of different isolates, the second derivative FTIR spectra in the carbohydrate adsorption region, as standardized by the manufacturer, were normalized. Hierarchical clustering (HCA) and principal component analysis of the normalized second derivative of each of the isolates were performed using Client Software v3.0 (Bruker Daltonik, USA). Standardization of media type, temperature, and incubation time allows for interpretation of specimens processed at different times. Following specimen preparation, the average time for result is 2 h. Whole genome sequencing (WGS) was utilized to assess the validity of spectral data analysis and not as a strict comparator method.

## Results

### Two-step clinical surveillance diagnostic algorithm *C. auris*

#### *C. auris* surveillance PCR assay

**Figure 1:**
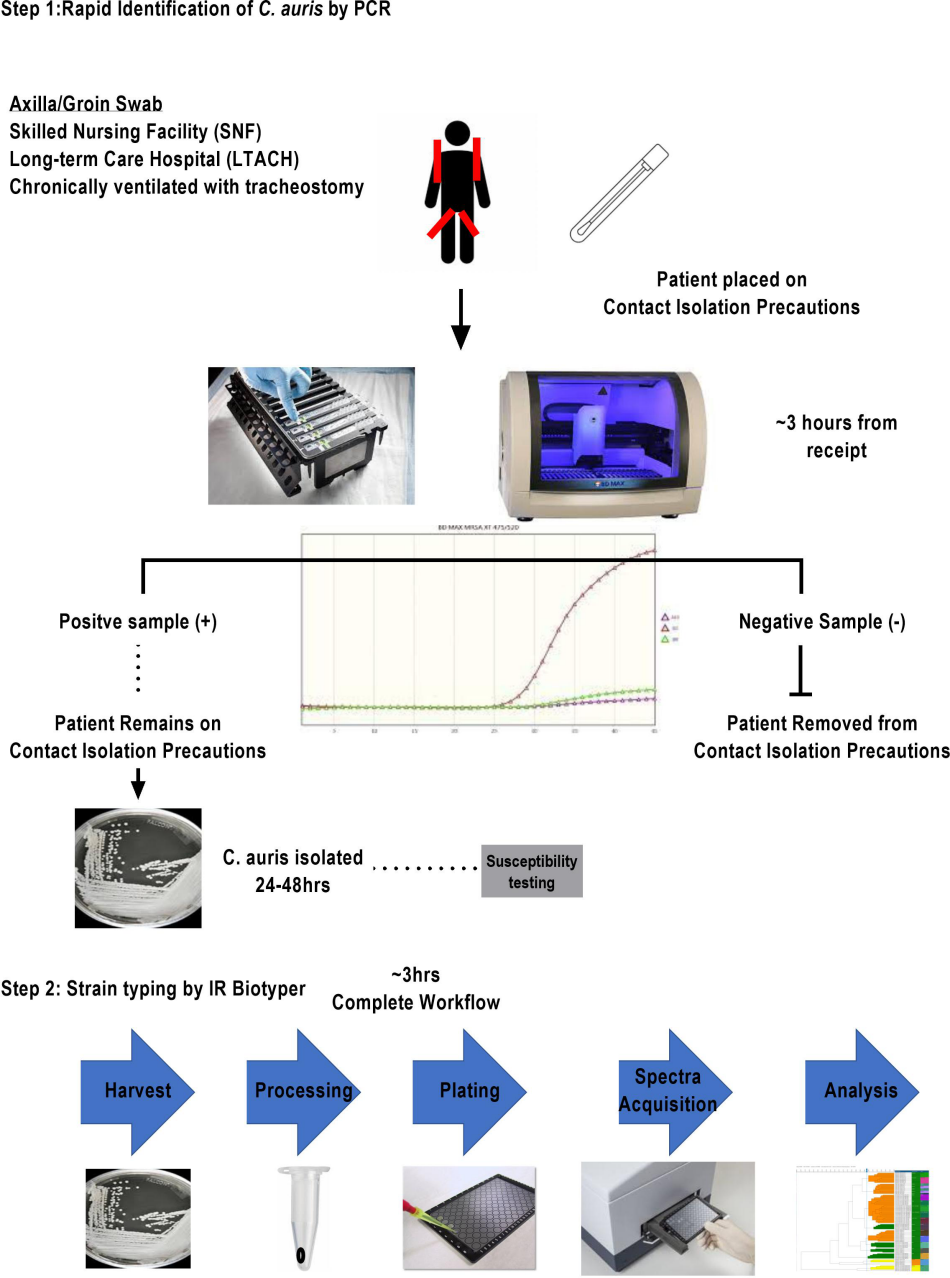
Two-tier clinical surveillance diagnostic algorithm for the detection of *C. auris.* This two-step procedure illustrated includes a rapid identification qualitative RT-PCR with an approximate turnaround time of 3 h for at-risk patient population meeting the criteria of transferring from SNF and LTACH or being chronically ventilated with tracheostomy. Any identified PCR-positive specimens are reflexed to fungal culture for isolation of organism. Once a pure culture *C. auris* is obtained, the laboratory sets up a susceptibility panel and a second pure culture isolate is prepared for strain typing.

While the COVID-19 pandemic was impacting the operations in microbiology laboratories, *C. auris* was appearing in medical facilities as an emerging nosocomial threat. The development, maintenance, and funding of a *C. auris* surveillance and outbreak investigation program was necessary for the control of *C. auris* mitigation within the hospital setting. The microbiology laboratory developed a two-step surveillance program to first detect patients colonized with *C. auris* and then provide nosocomial transmission information from analysis of the cultured yeast (**Figure 1**). With the cooperation of our local public health department and our epidemiology team, criteria were established for the patient population needing surveillance. All had one or more of the following risk factors: admission from skilled nursing facility (SNF), long-term care hospital (LTACH), and patients that are chronically ventilated or with a tracheostomy. Daily PCR testing runs were performed from axilla/groin specimens collected from patients admitted with risk factors using an Eswab (BD, Inc., USA) and analyzed using the BioGX Candida auris rt-PCR reagents for the BD™ MAX open testing system. The limit of detection (LOD) was established using quantitative *C. auris* template control with a known total concentration of 10^5^ copies/ml. Assay sensitivity was found to be set at 100 copies/ml using a total of 40 amplification cycles as set forth and optimized by the manufacturer based on the utilization of the BD™ Extk DNA-3 extraction protocol. On the basis of the established analytical sensitivity, a cycle threshold (Ct) value of ≤ 36 was reported as detected for *C. auris* and a Ct value of greater than 36 would need chart review by the medical director or designee. The coefficient variance was found to be less than 8% when Ct values across different concentrations were measured and compared. Consistent range of Ct values was established, making the assay reliable and highly reproducible. Utilization of CDC and FDA Antibiotic Resistance Isolate Bank *Candida auris* Panel was utilized to establish analytical specificity. The panel consisted of confirmed *C. auris* strains as well as close and distant genetic strains such as *Candida duobushaemulonii*, *C. haemulonii*, *C. krusei*, *C. lusitaniae*, and *Saccharomyces cerevisiae.* It was found that the qualitative RT-PCR was highly specific and sensitive (**Table 1**). Over the past year, we have successfully screened a total of 716 high-risk patients with a positivity rate of 3.9% (n = 28). Taken together, the data support the findings put forth by Leach and colleagues.

**Table 1:** CDC and FDA AMR *C. auris* rt-PCR data showing high specificity and sensitivity.

CDC ARBank#	Organism name	Clade	Specimen name	BD MAX BioGX *C. auris* Result		Agreement (Y/N)
				*C. auris POS (+)*	*C. auris NEG (-)*	
381	*Candida auris*	East Asia	CAU 1	x		Y
382	*Candida auris*	South Asia	CAU 2	x		Y
383	*Candida auris*	Africa	CAU 3	x		Y
384	*Candida auris*	Africa	CAU 4	x		Y
385	*Candida auris*	South America	CAU 5	x		Y
386	*Candida auris*	South America	CAU 6	x		Y
387	*Candida auris*	South Asia	CAU 7	x		Y
388	*Candida auris*	South Asia	CAU 8	x		Y
389	*Candida auris*	South Asia	CAU 9	x		Y
390	*Candida auris*	South Asia	CAU 10	x		Y
391	*Candida duobushaemulonii*	NA	391		x	Y
392	*Candida duobushaemulonii*	NA	392		x	Y
393	*Candida haemulonii*	NA	393		x	Y
394	*Candida duobushaemulonii*	NA	394		x	Y
396	*Kodameae ohmeri*	NA	396		x	Y
397	*Candida krusei*	NA	397		x	Y
398	*Candida lusitaniae*	NA	398		x	Y
399	*Saccharomyces cerevisiae*	NA	399		x	Y
400	*Saccharomyces cerevisiae*	NA	400		x	Y
931	*Candida auris*	South American	CAU21	x		Y
932	*Candida haemulonii*	NA	932		x	Y
1097	*Candida auris*	Iranian	CAU23	x		Y
1099	*Candida auris*	Iranian	CAU24	x		Y
1100	*Candida auris*	Iranian	CAU25	x		Y
1101	*Candida auris*	East Asian	CAU26	x		Y
1102	*Candida auris*	African	CAU27	x		Y
1103	*Candida auris*	African	CAU28	x		Y
1104	*Candida auris*	South American	CAU29	x		Y
1105	*Candida auris*	South American	CAU30	x		Y
	*Concordance*			100%		

NA, Not Applicable.

#### Fourier transform infrared Biotyper for strain typing

**Figure 2:**
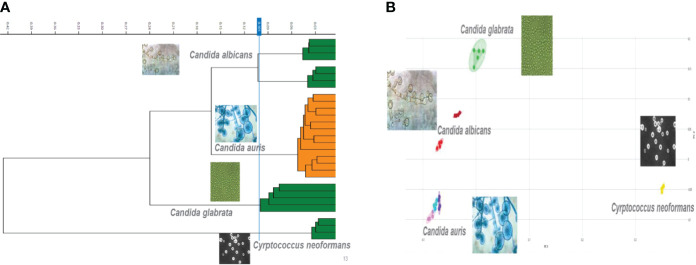
Discriminatory power of the IR Biotyper for the differentiation of yeast isolates. **(A)** Dendogram illustrating hierarchical cluster analysis (HCA) and principal component analysis **(B)** of clinically relevant yeast species including *Candida albicans*, *Candida glabrata*, *Candida auris*, and *Cryptococcus neoformans.* In the scatterplot, each isolate is displayed by a different color, and each geometric form represents a single spectrum.

**Figure 3:**
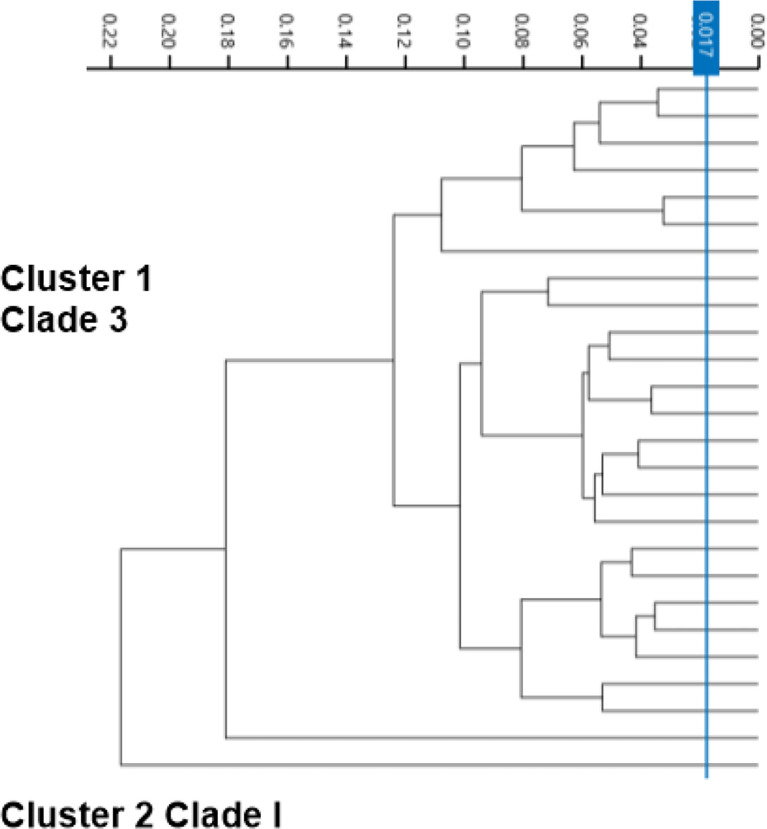
Spectra analysis using the IR Biotyper for strain typing. Dendogram illustrating hierarchical cluster analysis (HCA) of identified *C. auris* strains from two affiliated hospitals over a 1-year period showing two distinct significant clusters. Cluster 1 is *C. auris* isolates belonging to African lineage (Clade III), major circulating strain within the Los Angeles area. Cluster 2 was identified to belong to South Asian Lineage (Clade I).

The second tier of our *C. auris* surveillance program was the ability to actively perform strain typing of all *C. auris* strains in real time. Whole genome typing data were used to assess the validity of the spectra analysis acquired by the IR Biotyper. First, we wanted to test the discriminatory power of the IR Biotyper among different clinically relevant yeast species, including *Candida albicans*, *Candida glabrata*, *Candida auris*, and *Cryptococcus neoformans.* Hierarchical cluster and principal component analysis allowed for the successful differentiation of different yeast isolates, which formed distinct clusters both at the genus and species levels (**Figure 2**). To assess the variability and similarity among the identified *Candida auris* isolates (n = 28), spectra acquisition (n = 420) was acquired by the IR Biotyper spectrometer, and its normalized second derivative was analyzed using Client Software v3.0. Whole genome single-nucleotide polymorphism (SNP)–based phylogenetic analysis of the *C. auris* sequenced genomes was used as reference for the IR-based spectrum typing method. Although these two platforms are measuring different output reads, there were clonal relationships that were found to be significant and were confirmed by both SNP-based phylogenetic and IR-based spectrum typing analysis. K-mer SNP analysis was conducted on the 28 sequenced genomes using previously *C. auris* sequences available on GenBank (Chow et al., 2020). This analysis showed assignment of the 28 C*. auris* isolates into two distinct lineages. Most of the *C. auris* isolates (n = 27) were closely related to African lineage (Clade III) apart from one *C. auris* isolate that was found to be genetically distinct. This single-isolate cluster was closely related to a *C. auris* isolate from the South Asian lineage (Clade I). IR-based spectrum typing analysis of all 28 C*. auris* isolates consistently confirmed the separation of the two specified clusters: Cluster 1, demonstrating isolates belonging to the African lineage, and Cluster 2, single-isolate cluster belonging to South Asian Clade (**Figure 3**). Further phylogenetic k-mer SNP analysis of *C. auris* isolates in Cluster 1 to its closest ancestral relative showed less than 30-SNP difference among the strains, which contained isolates from both the main hospital and its affiliated clinical center. IR spectra typing analysis distance matrix supported the WGS reference data that the isolates found in Cluster 1 were from both affiliated hospitals. Together, these data demonstrate that the genetic background of isolates from Cluster 1 between both hospitals are very similar. Given the fact that the evolutionary rate of *C. auris* is approximately 5.7 × 10^−5^ substitutions per site per year and that the majority of circulating *C. auris* strains are genetically very similar suggests that population genetics are geospatial specific in origin with subsequent spread, making transmission within a hospital setting very hard to definitively conclude without the support of additional epidemiological evidence. Collectively, IR spectrum typing analysis performed on the IR Biotyper spectrometer was able to successfully differentiate clinically relevant yeast species both at the genus and species levels as well as differentiate between distinctly different clusters belonging to two different lineages of *C. auris* strains.

## Discussion

*Candida auris* has emerged as a critical multi-drug resistant pathogen having significant clinical impact. Dealing with a silent critical pathogen such as *Candida auris* amid a COVID-19 pandemic requires immediate action from the clinical and laboratory team. Here, we describe a two-tier *Candida auris* surveillance diagnostic algorithm composed of a surveillance rt-PCR for the rapid detection of the organism complemented by a strain typing platform to dissect clonal relatedness in real-time. The BioGX *Candida auris* rt-PCR reagents for the BD™ MAX direct from specimen testing were found to have great sensitivity, specificity, and reproducibility in the rapid detection of *C. auris*. It has allowed the clinical laboratory to work alongside our epidemiology team to identify cases and implement infection control measures to prevent transmission within the hospital setting. The *C. auris* rt-PCR has provided rapid results with minimal hands-on time within 2.5 h of setup, which is considerably more rapid than the standard culture method that can take anywhere from 48 to 96 h to isolate and identify. The assay was highly sensitive with LOD of 100 copies/ml, associated with a Ct value of 36. Most importantly, the BioGX RT-PCR was found to be highly specific (100%) having no cross-reactivity with genetically similar yeast relatives such as *Candida duobushaemulonii*, *C. haemulonii*, *C. krusei*, *C. lusitaniae*, and *Saccharomyces cerevisiae.* Our RT-PCR was able to successfully detect *C. auris* isolates from different clades (CDC strains), showing it will be able to detect new genetic variants associated specifically within a lineage clade. This is important because the WGS data have shown us that there is geospatial genetic variation within a regional population as seen in the Los Angeles area and across the country (Chybowska et al., 2020).

The second part in controlling the spread of *Candida auris* within the clinical setting is having the ability to conduct outbreak investigations and tracking any new variant strain circulation. The IR Biotyper has proven to show its potential in microbial strain typing when using WGS as a reference method and validity. When it comes to IR Biotyper application of yeast isolate differentiation, data are limited, and, to our knowledge, this is the first report of its application for the differentiation of different yeast species, including *Canida auris*. It was found that the IR Biotyper was able to allow for the successful discrimination of various yeast isolates, which included *Candida albicans*, *Candida glabrata*, *Candida auris*, and *Cryptococcus neoformans.* Furthermore, IR spectrum typing method distributed the processed *C. auris* isolates into two different lineages, as supported by phylogenetic genomic analysis. It was able to separate the isolates into two distinct lineages with the majority of the circulating strains belonging to common ancestral strain belonging to African lineage (Clade III) and a single-isolate cluster to South Asian lineage (Clade I), which furthers support the running hypothesis of Los Angeles County *C. auris* strains primarily belonging to African lineage (Price et al., 2021). The IR Biotyper is a great alternative for moderate to high-complexity laboratories looking for strain typing platform that is moderately priced, technically friendly with post-analysis ease. The IR Biotyper provides spectra within the 2 h of processing, and it does not require extensive prior knowledge of FTIR technology. Since the IR Biotyper is a phenotypic method, it is important to standardize, especially for yeast, the growth conditions such as temperature and growth media to reduce technical variability (Szekely et al., 2019). In order to ensure a high level of technical reproducibility, all samples were processed in quintuplicates. The new IR Biotyper Software v3.0 allows for AI learning capability. This will allow a laboratory to create its own patient database and identify any trends or shifts in the genetic lineage of the yeast in real-time.

In summary, *C. auris* surveillance PCR for detection of colonized patients paired with the Bruker IR Biotyper for fungal strain typing provides a two-step algorithm that has proven to be effective in the control of nosocomial spread of *C. auris* transmission in our hospital amid the COVID-19 pandemic.

## Data availability statement

The original contributions presented in the study are included in the article/supplementary material. Further inquiries can be directed to the corresponding author.

## Author contributions

All authors listed have made a substantial, direct, and intellectual contribution to the work, and approved it for publication.

## Funding

Supported in part by the Special Pathogens Grant (235461) from California Department of Public Health. The contents are solelythe responsibility of the authors and do not necessarilyrepresent the official views of the CDPH.

## Conflict of interest

The authors declare that the research was conducted in the absence of any commercial or financial relationships that could be construed as a potential conflict of interest.

## Publisher’s note

All claims expressed in this article are solely those of the authors and do not necessarily represent those of their affiliated organizations, or those of the publisher, the editors and the reviewers. Any product that may be evaluated in this article, or claim that may be made by its manufacturer, is not guaranteed or endorsed by the publisher.
